# Single-Center Experience With Minimally Invasive Approaches From Sternotomy to Thoracoscopy: Assessing the Learning Curve and Benefits

**DOI:** 10.7759/cureus.101766

**Published:** 2026-01-18

**Authors:** Mohamed H Elsayed, Ahmed Daoud, Wael Hassanein, Amr A Rayan

**Affiliations:** 1 Cardiothoracic Surgery, University of Alexandria, Alexandria, EGY

**Keywords:** cardiopulmonary bypass, full sternotomy, surgical replacement of valve, transesophageal echo, video-assisted thoracoscopic surgery (vats)

## Abstract

Background

Cardiac surgery is increasingly shifting from traditional full sternotomy (FS) towards minimally invasive cardiac surgery (MICS), driven by evidence of benefits like reduced trauma, faster recovery, and shorter hospital stays. However, the adoption of MICS is hindered by a significant learning curve, technical complexity, and the need for specialized team training and equipment. This study aimed to evaluate the initial outcomes and safety of a newly established MICS program by comparing its first cases with concurrent FS procedures.

Results

A single-center prospective cohort study was conducted from November 2024 to April 2025, including 98 patients undergoing primary, elective single-valve surgery. Patients were allocated to either the MICS group (n=30), representing the program's initial experience, or the FS group (n=68). The FS group, which had a higher preoperative risk profile and more complex procedures, served as the control. The MICS group demonstrated significantly longer operative and cardiopulmonary bypass times (6.56 vs. 4.87 hours; 122.93 vs. 92.66 minutes, p<0.001). However, MICS patients experienced substantially less blood loss (288.3 vs. 592.7 mL, p<0.001) and lower transfusion requirements. Postoperatively, the MICS group had lower pain scores, faster mobilization (1.23 vs. 2.31 days, p<0.001), shorter hospital stays (6.20 vs. 7.74 days, p=0.004), and a markedly quicker return to normal activity (2.50 vs. 10.49 weeks, p<0.001). A significant increase in phrenic nerve palsy was observed in the MICS group (16.7% vs. 0%, p=0.001). Crucially, there were no significant differences in mortality, stroke, or reoperation for bleeding.

Conclusion

Despite a clear learning curve, as evidenced by longer procedure times and a higher rate of procedure-specific complications such as phrenic nerve palsy, the initial implementation of a minimally invasive program was safe and conferred significant patient benefits, including enhanced recovery and superior patient-reported outcomes. Prudent patient selection and a structured team-based approach are essential for navigating the learning phase successfully. These findings support the feasibility of establishing such a program without compromising fundamental patient safety.

## Introduction

Cardiac surgery is undergoing a major transformation, gradually shifting from the traditional full sternotomy (FS), long considered the standard access, to minimally invasive cardiac surgery (MICS). This transition is driven by a growing body of evidence demonstrating clear patient-centered benefits. Compared with FS, MICS approaches, such as right mini-thoracotomy and upper mini-sternotomy, are associated with reduced surgical trauma, lower postoperative pain, shorter hospital stays, and a faster return to normal daily activities and productivity [1-3]. These advantages increasingly shape patient expectations and have become important factors in postoperative recovery pathways.

The physiological basis for these benefits is well described. MICS preserves respiratory mechanics, avoids sternal disruption, reduces wound-related complications, and is generally associated with a lower inflammatory response, decreased perioperative pain, and a reported better recovery [4,5]. Lower transfusion requirements, which themselves correlate with reduced risks of infection, renal dysfunction, and mortality, represent another key advantage [6].

Despite these benefits, the adoption of MICS remains challenging. Operating through small incisions with long instruments and limited tactile feedback requires substantial technical adaptation. Surgeons must acquire new skills, including percutaneous femoral cannulation, endoscopic or video-assisted visualization, and suturing in a restricted field [7,8]. This phase is universally associated with a steep learning curve, reflected in longer operative and cardiopulmonary bypass (CPB) times during early experience. These factors can raise concern for patient safety, increase operative stress, and potentially affect operating room efficiency.

Successful implementation also depends on a dedicated, well-trained multidisciplinary team. Anesthesiologists must be proficient in transesophageal echocardiography, one-lung ventilation, and peripheral cannulation management. Perfusionists require expertise with alternative cannulation strategies, and scrub nurses must be familiar with specialized instrumentation and workflow. In addition, MICS programs demand significant institutional investment in equipment such as long-shafted instruments, videoscopes, and peripheral cannulation sets [9,10]. For many centers, especially those with limited resources, these requirements constitute major barriers to establishing a program.

The learning curve itself introduces psychological and ethical challenges. Early cases are often associated with prolonged operative times and may carry higher rates of approach-specific complications, such as phrenic nerve palsy with right mini-thoracotomy approaches [11,12]. Institutions must balance the temporary increase in technical complexity and risk during the initial phase against the well-established long-term benefits of a mature program [13-15].

Given these challenges, there remains a need for transparent, real-world data describing the earliest phase of MICS adoption, including the difficulties encountered and the safety profile during the initial learning curve. This study aims to address this gap by examining outcomes of the first cases performed in a newly established MICS program compared with concurrent FS procedures. The objective is to provide a realistic assessment of the early learning period and offer practical insights for centers beginning their transition toward minimally invasive approaches.

## Materials and methods

This single-center, prospective cohort study included all consecutive adult patients who underwent elective primary valve surgery for a single-valve pathology at our institution between November 2024 and April 2025. Functional tricuspid regurgitation accompanying mitral valve disease was considered part of the same single-valve pathology when treated with concomitant tricuspid repair.

Patients were followed for a minimum of four months postoperatively. The surgical approach (MICS or conventional FS) was not randomized. Allocation was determined by the cardiac surgery team affiliated with the MICS program. Factors influencing selection included chest and cardiac anatomy (CT chest, transthoracic echocardiography), iliofemoral vessel caliber (added as a routine assessment during the study period), comorbidities and frailty, procedural complexity, and surgeon preference and level of MICS experience. This approach reflects real-world patient selection during the early phase of a MICS program.

Inclusion and exclusion criteria

The study included adult patients aged ≥18 years who underwent elective, first-time cardiac surgery or isolated single-valve surgery (aortic, mitral, or tricuspid). All patients undergoing MICS from the start of the program (November 2024 onward) were also included. The exclusion criteria were applied to minimize heterogeneity among cases and to avoid high-risk confounders. These comprised multiple concomitant procedures (e.g., CABG (coronary artery bypass grafting), multiple valve surgery, atrial fibrillation surgery), emergency or salvage surgery, redo cardiac surgery (any prior sternotomy or cardiac operation), and active endocarditis requiring complex reconstruction.

Surgical techniques

For MICS, all procedures were performed via a right mini-thoracotomy (5-7 cm). Key technical steps included establishing femoro-femoral CPB before the thoracic incision. Negative suction was applied to the cardiotomy reservoir. An additional SVC cannula is inserted via the neck for patients requiring tricuspid surgery or weighing >85 kg. Routine video assistance for exposure and suture visualization was utilized. Aortic occlusion using a transthoracic clamp, and lastly, Cor-Knot® devices were used for all valve sutures.

For FS, procedures were performed via a standard median sternotomy with central aortic cannulation and right atrial or bicaval venous cannulation as deemed appropriate. Conventional aortic cross-clamping and cardioplegia were performed in the regular fashion (crystalloid cardioplegia).

All intraoperative management (cardioplegia strategy, temperature protocols, and perfusion parameters) was standardized between groups.

Data collection and outcomes

All data were collected prospectively from the institutional cardiac surgery database and electronic medical records. Follow-up was performed through outpatient visits and anticoagulation clinic appointments, supplemented by telephone assessment when required.

Preoperative and intraoperative variables

Preoperative variables include demographics and comorbidities, EuroSCORE, echocardiographic measurements, and baseline laboratory values. Intraoperative variables include the type of valve procedure, cannulation strategy, cross-clamp time, CPB time, and total operative time.

Postoperative variables

Postoperative variables include duration of mechanical ventilation, ICU and total hospital length of stay, transfusion requirements, major complications (mortality, stroke, reoperation for bleeding, renal failure), and procedure-specific complications (e.g., phrenic nerve palsy, groin complications).

Follow-up outcomes at ≥4 months include readmission rates, mortality, pain scores (numerical rating scale, NRS), time to return to normal activity (patient self-report + structured follow-up questionnaire), and wound satisfaction (Likert scale).

Statistical analysis

Continuous variables are presented as mean ± standard deviation and were compared using Student’s t-test or the Mann-Whitney U test, depending on their distribution. Categorical variables are presented as numbers and percentages and were compared using the chi-square test or Fisher’s exact test, as appropriate. A two-sided p-value of <0.05 was considered statistically significant. All analyses were performed using IBM SPSS Statistics for Windows, Version 28 (Released 2021; IBM Corp., Armonk, New York).

Ethics approval

The study received approval from the Institutional Review Board (IRB) or Ethics Committee of the University of Alexandria (approval no. 00012098). All patients provided standard informed consent for surgery; no study-specific interventions were performed.

## Results

The study included 98 patients who underwent isolated primary valve surgery, comprising 30 patients in the MICS group and 68 in the FS group. All MICS procedures were performed by a single surgeon early in their MICS training, supported by a fixed surgical and anesthetic team from the start of the program.

Preoperative characteristics

Significant baseline differences were observed between the groups. The MICS cohort had a higher proportion of female patients (53.3% vs. 23.5%, p=0.005). The FS group demonstrated a higher overall risk profile (Table 1), including a higher BMI (26.7 vs. 24.1, p=0.009), lower ejection fraction (56.2% vs. 59.9%, p=0.009), higher prevalence of chronic obstructive pulmonary disease (COPD) (25.0% vs. 10.0%, p=0.048), and higher EuroSCORE II (1.3 ± 0.9 vs. 1.0 ± 0.3, p=0.027).

**Table 1:** Comparative Analysis: MICS (n=30) vs. FS (n=68) Comparative baseline demographics and clinical characteristics of patients undergoing minimally invasive cardiac surgery (MICS) versus full sternotomy (FS). The MICS group had a significantly higher ejection fraction, whereas the FS group had a higher BMI, a higher EuroSCORE II, and a higher prevalence of COPD. Values are presented as mean and standard deviation (SD). The p-value is considered significant if P ≤ 0.05. For continuous variables with normal distribution, independent t-tests were used. For continuous variables with a non-normal distribution, the non-parametric Mann-Whitney U test was employed. Chi-square/Fisher's Exact tests were used for the categorical variables to generate the p-values in this comparative table. BMI = Body mass index, COPD = Chronic obstructive pulmonary disease, EuroSCORE II = European System for Cardiac Operative Risk Evaluation II. Predicts risk of in-hospital mortality after major cardiac surgery, FS = Full sternotomy, MICS = Minimally invasive cardiac surgery

Parameter	MICS Group (Mean ± SD)	FS Group (Mean ± SD)	p-value	Significance
Age (Years)	45.5 ± 12.4	47.8 ± 12.3	0.382	Not Significant
Sex (Females)	53.3%	23.5%	< 0.001	Significant
BMI	24.1 ± 3.9	26.7 ± 6.3	0.009	Higher in FS
Ejection Fraction (EF, %)	59.9 ± 5.4	56.2 ± 7.4	0.009	Higher in MICS
Euroscore II	0.83 ± 0.26	1.05 ± 0.71	0.039	Higher in FS
Creatinine (mg/dL)	1.40 ± 1.58	1.16 ± 0.25	0.303	Not Significant
Categorical Parameter	MICS Group (%)	FS Group (%)	p-value	Significance
COPD	10.0% (3/30)	25.0% (17/68)	0.048	Higher in FS
Diabetes	3.3% (1/30)	8.8% (6/68)	0.678	Not Significant
Hypertension	70.0% (21/30)	86.8% (59/68)	0.193	Not Significant

The FS approach was used more frequently for combined valve procedures, such as mitral-tricuspid surgery (25.0% vs. 3.3%, p=0.009), as shown in Table 2. MICS was used predominantly for mitral valve replacement (95.0% of mitral cases), while FS included a more balanced distribution of repairs and replacements (53.1% vs. 46.9%, p<0.001).

**Table 2:** Statistical Analysis of Type of Procedures: MICS vs. FS Distribution and statistical comparison of the primary surgical procedures performed. The MICS group had a significantly higher rate of mitral valve replacement, while the FS group had a higher rate of combined mitral and tricuspid procedures and mitral valve repair. The p-value is considered significant if P ≤ 0.05. FS = Full sternotomy, MICS = Minimally invasive cardiac surgery

Procedure Type	MICS Group (n=30)	FS Group (n=68)	Statistical Test	p-value	Significance
Mitral Procedures	20 (66.7%)	49 (72.1%)	Chi-square	0.582	Not Significant
Aortic Procedures	5 (16.7%)	14 (20.6%)	Chi-square	0.637	Not Significant
Tricuspid Procedures	3 (10.0%)	4 (5.9%)	Fisher's Exact	0.427	Not Significant
Cardiac Tumor	2 (6.7%)	1 (1.5%)	Fisher's Exact	0.171	Not Significant
Mitral + Tricuspid	1 (3.3%)	17 (25.0%)	Fisher's Exact	0.009	Significant
Mitral Valve Replacement	19/20 (95.0%)	23/49 (46.9%)	Fisher's Exact	<0.001	Significant
Mitral Valve Repair	1/20 (5.0%)	26/49 (53.1%)	Fisher's Exact	<0.001	Significant

Intraoperative outcomes

A clear trade-off was observed between the approaches. Operative time was significantly longer in the MICS group (6.56 hours vs. 4.87 hours, p<0.001), as was CPB time (122.93 vs. 92.66 minutes, p<0.001), as shown in Table 3. However, mean operative blood loss was significantly lower in the MICS group (288.3 mL vs. 592.7 mL, p<0.001) (Table 4). Consequently, MICS patients required fewer transfusions of packed red blood cells (1.17 vs. 2.31 units, p<0.001) and fresh frozen plasma (0.83 vs. 2.40 units, p<0.001).

**Table 3:** Comparative Analysis of Operative Time Comparison of key operative time metrics. Total operative time and cardiopulmonary bypass (CPB) time were significantly longer in the MICS group, while aortic cross-clamp times were similar. Values are presented as mean and standard deviation (SD). The p-value is considered significant if P ≤ 0.05. MICS = Minimally invasive cardiac surgery

Parameter	MICS Group (Incision=1) (n=30)	Full Sternotomy Group (Incision=4) (n=68)	Statistical Test	p-value
Operative Time (hrs, Mean ± SD)	6.56 ± 1.07	4.87 ± 0.83	Mann-Whitney U	<0.001
CPB Time (min, Mean ± SD)	122.93 ± 39.84	92.66 ± 17.62	Mann-Whitney U	<0.001
Cross Clamp Time (min, Mean ± SD)	67.93 ± 24.60	65.71 ± 16.31	Mann-Whitney U	0.869

**Table 4:** Bleeding, Transfusions, and Reoperation: MICS vs. FS Postoperative bleeding and transfusion requirements. The MICS group demonstrated significantly lower intraoperative blood loss and required fewer transfusions of packed red blood cells and fresh frozen plasma, despite a similar reoperation rate for bleeding. Values are presented as mean and standard deviation (SD). The p-value is considered significant if P ≤ 0.05. FS = Full sternotomy, MICS = Minimally invasive cardiac surgery, FFP = Fresh frozen plasma

Parameter	MICS Group (n=30)	FS Group (n=68)	Statistical Test	p-value	Significance
Operative Bleeding (mL)	288.3 ± 132.2	592.7 ± 268.8	Mann-Whitney U	<0.001	Significant
Reoperation for Bleeding (ReTx)	3.3% (1/30)	7.4% (5/68)	Fisher's Exact	0.665	Not Significant
Blood Transfusions (Units)	1.17 ± 0.87	2.31 ± 1.14	Mann-Whitney U	<0.001	Significant
FFP Transfusions (Units)	0.83 ± 0.75	2.40 ± 1.14	Mann-Whitney U	<0.001	Significant

Postoperative outcomes

Postoperative recovery parameters generally favored the MICS approach. MICS patients reported lower pain scores (5.50 vs. 6.59, p<0.001), mobilized earlier (1.23 vs. 2.31 days, p<0.001), and had a shorter total hospital stay (6.20 vs. 7.74 days, p=0.004), as shown in Table 5. They returned to normal activity significantly sooner (2.50 vs. 10.49 weeks, p<0.001) and expressed higher wound satisfaction (4.50 vs. 3.10, p<0.001).

**Table 5:** Postoperative Recovery Metrics: MICS vs. FS Postoperative recovery indicators. Time to patient mobilization and total hospital stay were significantly shorter in the MICS group, while mechanical ventilation time and ICU stay were comparable. MICS = Minimally invasive cardiac surgery, FS = Full sternotomy

Parameter	MICS Group (n=30)	FS Group (n=68)	Statistical Test	p-value	Significance
Ventilation Time (hours)	5.07 ± 2.13	4.73 ± 1.60	Mann-Whitney U	0.394	Not Significant
ICU Stay (days)	3.57 ± 1.38	3.40 ± 1.27	Mann-Whitney U	0.464	Not Significant
Mobilization (days)	1.23 ± 0.43	2.31 ± 0.87	Mann-Whitney U	<0.001	Significant
Hospital Stay (days)	6.20 ± 2.13	7.74 ± 2.64	Mann-Whitney U	0.004	Significant

However, the MICS group experienced a higher rate of phrenic nerve palsy (16.7% vs. 0.0%, p=0.001). All rehospitalizations in the MICS cohort (6.7%) were due to groin-related complications associated with femoral access.

There were no significant differences between the groups in mortality, stroke, reoperation for bleeding, or postoperative endocarditis.

## Discussion

This single-center prospective study presents the real-world outcomes of establishing a MICS program, beginning with the first patients treated using a true thoracotomy-based approach. By comparing these initial cases with concurrently performed FS procedures, the study offers practical insight into the challenges, risks, and benefits encountered during the earliest phase of the MICS learning curve.

Although the advantages of MICS are well recognized, its global adoption has remained slow. Median sternotomy continues to be the predominant approach due to the substantial technical learning curve and the institutional investment required [16]. For this reason, early-experience reports remain important, providing essential guidance for new programs. The literature consistently emphasizes that successful implementation requires not only the acquisition of new surgical skills but also a coordinated, multidisciplinary effort including anesthesia, perfusion, and nursing teams [17]. Contemporary data suggest that proficiency is generally achieved after approximately 50-100 cases, during which outcomes progressively approach those of established high-volume centers [18]. Thus, early-experience studies continue to play an important role in helping new centers navigate this complex process.

Our results align with the experiences reported in the literature. The prolonged operative and CPB times in the MICS group are characteristic of the early learning curve (Table 3) and reflect the technical complexity of adapting to limited-incision surgery [13]. An important factor in interpreting these findings is the deliberate and necessary use of careful patient selection during the early phase of program development. As recommended in multiple reports [19], we selected cases with favorable anatomy and lower technical complexity for MICS. This is reflected in the predominance of straightforward mitral replacements, larger aortic annuli, and the allocation of more complex cases, such as mitral repairs requiring concomitant tricuspid intervention, to the FS cohort. Such early selection bias is widely accepted as essential to maintaining safety while skills and workflows are developing.

Despite the technical challenges, cross-clamp times remained comparable between the groups. This may be partly attributable to the use of facilitating technologies, such as the Cor-Knot® system, which expedites suture tying and may partially offset early inefficiencies [17,18]. The incorporation of supportive technologies has been shown to accelerate the MICS learning curve and promote consistent outcomes. Despite observing a significant difference between the two groups in overall operative time and total CPB favoring the FS group (Table 3), we also noted a desirable benefit of MICS, with an overall reduction in postoperative bleeding and blood and blood product transfusion requirements (Table 4).

The increased occurrence of phrenic nerve palsy in the MICS group indicates a recognized, procedure-specific complication linked to right mini-thoracotomy in early practice [12]. While these cases did not significantly alter postoperative clinical recovery, they highlight the importance of diaphragmatic protection strategies during exposure and retraction. Importantly, major outcomes, including mortality, stroke, and reoperation for bleeding, did not differ significantly between the two groups, supporting the safety of introducing MICS even during the learning phase, as reported in other early-experience studies [18].

Postoperative recovery metrics favored the MICS approach, with earlier mobilization, shorter hospital stays, quicker returns to normal activity, and higher patient satisfaction regarding pain and wound appearance (Table 5). These findings align with established MICS literature [1,2]. However, they must be interpreted with caution, as the FS group represented a higher-risk cohort with a greater proportion of complex pathology, which may have magnified the recovery differences seen in this study.

A further indication of the presence of patient selection between the surgical groups. Patients undergoing MICS had a significantly larger aortic annulus (p = 0.0015; Table 6). Despite both groups having similar preoperative left atrial sizes (Table 7), patients were more likely to receive a concomitant left atrial appendage closure in the FS group (0% in MICS vs. 11.8% in the FS group) (Table 8). This pattern indicates that the MICS cohort was selected for anatomically simpler cases, favoring less complex interventions suitable for a minimal-access approach.

**Table 6:** MICS vs. FS Aortic Valve Replacement (AVR) Annulus Size Comparison of implanted prosthetic valve sizes in patients undergoing isolated aortic valve replacement (AVR). A larger mean annulus size was observed in the MICS group. Values are presented as mean and standard deviation (SD). The p-value (0.0015, from Welch's t-test) indicates a statistically significant difference in mean aortic annulus size between the MICS and FS groups. FS = Full sternotomy, MICS = Minimally invasive cardiac surgery

Group	n	Mean Annulus Size (mm)	Standard Deviation	Range (mm)	p-value
MICS	8	23.88	± 1.36	22–25	0.0015
Full Sternotomy	13	21.38	± 1.66	19–24	

**Table 7:** Left Atrial Size for Mitral Procedures: MICS vs. FS Preoperative left atrial dimensions in patients undergoing mitral valve procedures. No statistically significant difference was found between the surgical groups. Values are presented as mean and standard deviation (SD). MICS = Minimally invasive cardiac surgery, FS = Full sternotomy

Group	n	Mean Left Atrial Size (mm)	Standard Deviation	Range (mm)
MICS	25	58.00	± 7.01	45–75
Full Sternotomy	41	56.05	± 10.24	35–74

**Table 8:** Left Atrial Appendage Closure (LAAC): MICS vs. FS Incidence of left atrial appendage closure (LAAC) concomitant with other cardiac procedures. The procedure was performed exclusively in a subset of the FS group. MICS = Minimally invasive cardiac surgery, FS = Full sternotomy

Group	N	LAAC Performed (n)	LAAC Performed (%)	No LAAC (n)	No LAAC (%)
MICS	30	0	0.0%	30	100.0%
Full Sternotomy	68	8	11.8%	60	88.2%

MICS unfortunately carries a risk of conversion to a full incision [20]. Our study also provided valuable insights into conversion management. Three patients required intraoperative conversion to sternotomy. Two conversions resulted from unfavorable anatomical factors that became evident intraoperatively, highlighting the critical importance of respecting anatomical limitations and maintaining a low threshold for conversion early in program development. The third conversion resulted from femoral artery bleeding during reperfusion, underscoring the importance of meticulous attention to cannulation and line management.

Additional lessons emerged from preoperative assessment. Three scheduled MICS cases were transitioned to sternotomy before thoracic incision. In two cases, inadequate femoral vessel caliber prompted abandonment of peripheral cannulation, leading to the adoption of routine preoperative femoral Doppler ultrasonography. A third case involved tricuspid surgery complicated by dense pleural adhesions, discovered on initial thoracoscopic inspection. These experiences reinforce the importance of detailed preoperative imaging and a stepwise, evaluative approach to access and exposure.

Study limitations

This study has several limitations. The non-randomized design introduces intentional selection bias, which, although necessary to ensure safety during program initiation, limits the comparability of outcomes between the two groups and may exaggerate recovery benefits. The sample size, particularly of the MICS cohort, is small and underpowered for detecting differences in uncommon complications. Furthermore, the results represent the early experience of a single surgeon and institutional team, which may limit generalizability to other centers with different structures or experience levels. Extending the cohort beyond the first 30 cases would have diluted the early-learning-curve focus that was central to this study's aim.

## Conclusions

The analysis of our new MICS program confirms the initial challenges well-documented in the literature, including longer procedure times and a trend toward specific complications. However, it demonstrates that through prudent case selection, the adoption of facilitating technologies, and strict preoperative and operative pathways, these challenges could be rewarded by significant and immediate patient benefits. This should encourage other institutions to undertake the journey of establishing a MICS program, providing a realistic expectation that even the initial learning phase can be accomplished safely when carefully applied while delivering meaningful improvements in patient care and outcomes.

We also find it of utmost importance to encourage surgeons to swiftly resort to a generous thoracotomy if found or suspected to be necessary. Deeming a patient unsuitable for thoracoscopy after induction of anesthesia should not be a surprise either, and hence, the thoracotomy incision should always be made after a systematic surgical approach to ensure the validity of a MICS procedure with the patient.
